# Efficient gene targeting in mouse zygotes mediated by CRISPR/Cas9-protein

**DOI:** 10.1007/s11248-016-9998-5

**Published:** 2016-11-30

**Authors:** Chris J. Jung, Junli Zhang, Elizabeth Trenchard, Kent C. Lloyd, David B. West, Barry Rosen, Pieter J. de Jong

**Affiliations:** 1 University of California, San Francisco Benioff Children’s Hospital Oakland Research Institute, Oakland, CA 94609 USA; 2 Gladstone Institutes, San Francisco, CA 94158 USA; 3 Wellcome Trust Sanger Institute, Cambridge, CB10 1SA UK; 4 Mouse Biology Program, University of California, Davis, CA 95618 USA

**Keywords:** CRISPR, Transgenic mouse model, Gene targeting

## Abstract

The CRISPR/Cas9 system has rapidly advanced targeted genome editing technologies. However, its efficiency in targeting with constructs in mouse zygotes via homology directed repair (HDR) remains low. Here, we systematically explored optimal parameters for targeting constructs in mouse zygotes via HDR using mouse embryonic stem cells as a model system. We characterized several parameters, including single guide RNA cleavage activity and the length and symmetry of homology arms in the construct, and we compared the targeting efficiency between Cas9, Cas9nickase, and dCas9–FokI. We then applied the optimized conditions to zygotes, delivering Cas9 as either mRNA or protein. We found that Cas9 nucleo-protein complex promotes highly efficient, multiplexed targeting of circular constructs containing reporter genes and floxed exons. This approach allows for a one-step zygote injection procedure targeting multiple genes to generate conditional alleles via homologous recombination, and simultaneous knockout of corresponding genes in non-targeted alleles via non-homologous end joining.

**Electronic supplementary material:**

The online version of this article (doi:10.1007/s11248-016-9998-5) contains supplementary material, which is available to authorized users.

## Introduction

Technologies enabling efficient and precise genome editing render powerful tools for studying biology, and open new avenues for explorative endeavors in biomedicine and translational research. Until recently, genome engineering in cell and animal models relied on random mutagenesis, random insertion of transgenes, or inefficient targeting, which greatly limited scientific progress (Stanford et al. 2001; Yu and Bradley 2001; Austin et al. 2004; Gondo 2008). Over the past decade, genome editing technologies have undergone a rapid procession of improvements in efficiency and precision with the development of zinc finger nucleases (ZFNs) (Kim et al. 1996; Bibikova et al. 2003; Maeder et al. 2008), and transcription activator-like effector nucleases (TALENs) (Christian et al. 2010; Boch 2011; Cermak et al. 2011). These tools are based on customizable DNA binding modules attached to nucleases for targeted chromosome breaks. More recently, the clustered regularly interspaced short palindromic repeats (CRISPR) associated protein 9 (Cas9) has emerged with great potential. In contrast to ZFNs and TALENs, which depend on protein-DNA interactions, the CRISPR/Cas9 system is based on the principle of engineering a single guide RNA (sgRNA) for base pairing with complementary DNA sequences for site-specific cleavage by the associated Cas9 protein complex (Gaj et al. 2013; Mali et al. 2013a, b; Sander and Joung 2014; Jiang and Marraffini 2015).

The inherent simplicity and flexibility imbued in the CRISPR/Cas9 architecture has propelled the system as the ideal genome engineering tool (Horvath and Barrangou 2010; Marraffini and Sontheimer 2010; Jinek et al. 2012; Wiedenheft et al. 2012; Cong et al. 2013; Mali et al. 2013a, b). As such, the system has been particularly useful for applications aimed at direct or conditional knockout of gene functions. For example, reports have shown that stimulating the error-prone mechanism of non-homologous end joining (NHEJ) repair (Rouet et al. 1994) by the sgRNA:Cas9 complex induced DNA breaks can knockout gene function by creating indel mutations (Cho et al. 2013; Shen et al. 2013; Wang et al. 2013; Sung et al. 2014) and that injecting single-strand oligonucleotides (ssODNs) carrying loxP sequences or short tags into zygotes can generate conditional alleles (Yang et al. 2014; Yoshimi et al. 2014; Renaud et al. 2016). However, despite the growing body of literature supporting the ease with which transgenic animals can be generated with the CRISPR/Cas9 system, approaches based on NHEJ or genome modification using ssODNs, suffer from imprecise NHEJ dependent genome modification, or short cargo carrying capacity and trans allele effect.

While using constructs may overcome these limitations, their low targeting efficiency with the CRISPR/Cas9 system hinders robust high-throughput applications. To date, only a few reports have described methods to knock small constructs into mouse zygotes with the CRISPR/Cas9 system. For example, Yang et al. (2013) injected circular reporter plasmids (*Nanog*-mCherry or *Oct4*-GFP) carrying homology arm lengths between 2 and 4.5 kbp with a targeting efficiency of approximately 10%, while Chu et al. (2016) targeted the *Rosa26* locus using vectors carrying asymmetric homology arm lengths between 1 and 4 kbp with a targeting efficiency of 0–20%. Moreover, Aida et al. (2015) described increased targeting efficiency of a circular EGFP-reporter vector with 2 kbp homology arms using Cas9 protein combined with chemically synthesized dual-crRNA:tracrRNA; however, their experiments were unsuccessful when using only the Cas9 protein. In contrast, Menoret et al. (2015) reported successful targeting using Cas9 protein and a linearized podocan-neoR cassette with 1 and 4.2 kbp asymmetric homology arms. Others reported success based on a single-targeted founder (F0) pup. Indeed, Wang et al. (2015) used a single-injection experiment to target 1 of 16 founder pups with a Cre cassette containing approximately 600 bp homology arms, and Lee and Lloyd (2014) used a single-injection in zygotes to successfully target 1 of 13 founder pups with a cassette containing a floxed critical exon with 1.9 kbp homology arms digested out of a circular vector. While these reports provide some insight, the scarcity of literature and the lack of protocol standards highlight a need to further optimize these methods and test their reliability.

Of particular relevance is the existence of more than 15,000 custom reporter vectors for conditional knockout are available to the public through repositories created by the Knockout Mouse Project (KOMP) Resource Center and the European Conditional Mouse Mutagenesis (EUCOMM) Center. This multi-center collaborative effort aims to ascribe the function of the entire mouse genome (Skarnes et al. 2011; Bradley et al. 2012). Despite these resources, the process of generating transgenic mouse models remains slow, because we lack an efficient and reliable method to target these constructs in mouse zygotes. As a result, many research facilities continue to rely on the traditional method of using ES cells to generate transgenic mouse models, which is cumbersome and inefficient (Capecchi 2005).

In this study, we aimed to develop an optimized condition for HDR mediated construct targeting in mouse zygotes using CRISPR/Cas9. First, we randomly selected constructs from the KOMP/EUCOMM repository that included small deletions of non-essential intronic sequences separating “critical exons” from upstream and downstream homology arms, which made them particularly useful for targeting studies. We then designed sgRNAs for these targets, which allowed correctly targeted genomic sites to resist further cutting by the Cas9:sgRNA complex. A representative sample of vectors was selected for in-depth analysis using embryonic stem (ES) cells as a model system for determining the optimal parameters for HDR-mediated targeting, which included comparing Cas9, Cas9nickase (Cas9n), and catalytically inactive Cas9 fused to FokI endonuclease (dCas9–FokI), and varying the length and symmetry of the homology arms. We applied the optimized conditions to zygotes and delivered Cas9 as either mRNA or protein to further hone and enhance the parameters. With our systematic approach for defining the optimal targeting conditions, we showed that Cas9 protein promotes an efficient multiplexed targeting of circular constructs containing reporter genes and floxed exons, and that this approach supports a one-step procedure to inject zygote to achieve both HDR mediated targeting of multiples genes and NHEJ induced deletion of gene function. Hence, we provide a blueprint describing an efficient and reliable method for CRISPR/Cas9 mediated construct targeting in mouse zygotes.

## Results

### Strategy for designing sgRNAs to mediate KOMP construct targeting in mouse ES cells

**Fig. 1:**
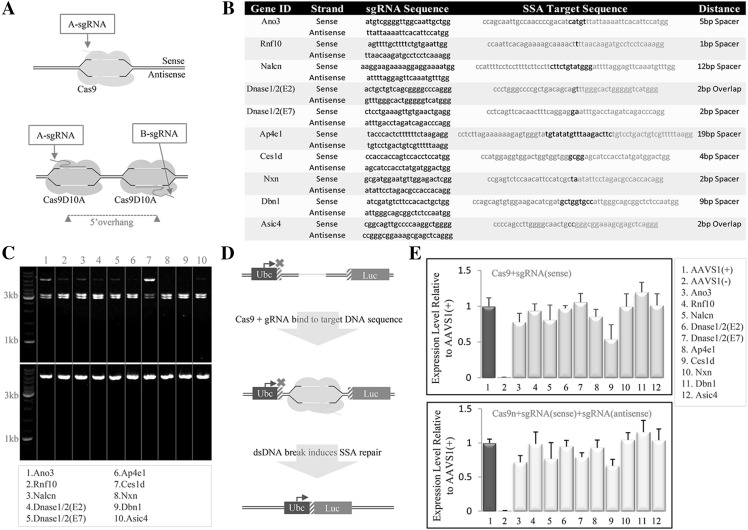
Strategy for CRISPR/Cas9(n) mediated KOMP construct targeting. **a** Diagram illustrating sgRNA-design strategy for use with Cas9 and/or Cas9n. The *top* illustration describes a single sgRNA:Cas9—complex strategy for inducing dsDNA cleavage. The* bottom* illustration describes a scenario in which a pair of sgRNA:Cas9n complexes are combined to induce nicks on opposite DNA strands, creating 5′ overhangs if the paired sgRNA:Cas9n complexes are not overlapping (the overlapping complexes form 3′ overhangs).* Green*, sgRNAs. **b** Table listing the sgRNAs selected for each gene in (**a**) (*first column*), the strand to which each sgRNA was designed to bind (*second column*), sgRNA sequences (*third column*), sgRNA-target sequences (*blue*, sgRNA target on the sense strand; *red*, sgRNA target on the antisense strand; *black*, spacer sequence between the two targets) (*forth column*), and spacer distance (*last column*). **c** Agarose analysis showing result of in vitro assay with Cas9 protein. **d** Schematic diagram illustrating the mechanism of the SSA assay. **e** *Bar graph* showing the SSA assay result relative to the *AAVS1* sgRNA. *Top graph*, result using Cas9; *bottom graph*, result using Cas9n. *AAVS1* (−) indicates the negative control, which is identical to AAVS1 (+) without sgRNA. (Color figure online)

To investigate whether CRISPR/Cas9(n) mediation enhances targeting of KOMP constructs in ES cells, we randomly selected vector constructs for nine genes from the KOMP repository (Fig. S1a–i). We designed two sgRNAs (A-sgRNA and B-sgRNA) as matched pairs for critical exon sequences in each of the nine genes, which would allow double-nicking with the *Streptococcal pyogenes* (Sp) Cas9 nickase mutant D10A (Ran et al. 2013), hereafter referred to as “Cas9n” (Fig. 1a, b). One of the sgRNAs (A-sgRNA) of each pair was used with the wild-type SpCas9 to generate blunt-ended DNA cuts. All sgRNAs were designed using the CRISPR guide–design tool developed by the Zhang lab at MIT (crispr.mit.edu) (Hsu et al. 2013).

### In vitro and ex vivo evaluation of sgRNA activity

To verify the efficiency of individual sgRNAs, we designed two assays to evaluate the capacity of selected sgRNAs to cleave dsDNA by wild-type Cas9 or double-nick opposite strands by Cas9n. First, we designed an in vitro approach in which the sgRNA target sequences were cloned into a plasmid that would be linearized at a unique restriction site in the vector backbone (Fig. S2a). Each linearized vector was incubated with the corresponding A-sgRNA and Cas9 protein, which produced two fragments when cleaved. Using this approach, we observed highly efficient dsDNA-cutting activity for all but two sgRNAs (i.e., *Ano3* and *Ces1d*), which showed relatively low efficiency (Fig. 1c). In the second assay, we designed an ex vivo approach to evaluate sgRNA activity in a mammalian cell culture system. Specifically, with a single-strand annealing (SSA) approach, we measured the capacity of single sgRNAs or matched sgRNA pairs to catalyze homologous recombination within a transfected plasmid, repairing a non-functional luciferase gene. The sgRNA target sequences were cloned in between direct repeats of the first 300 bp of the firefly luciferase open reading frame (ORF), with the upstream repeat having a Ubc promoter and the downstream 300 bp connected to the remaining ORF sequence. Cuts induced by Cas9 or matched nicks induced by Cas9n can stimulate homologous recombination between the repeats to generate a functional luciferase, measured by fluorescence (Fig. 1e). In this assay, the sgRNA target plasmid, expressing the sgRNA under the mammalian PGK promoter, and a Cas9- or Cas9n-expressing plasmid were co-transfected into T293 cells. Human *adeno*-*associated virus integration site 1* (*AAVS1*) sgRNA with a robust genome-editing capability was used as a positive control (Mali et al. 2013). The SSA assay revealed that all sgRNAs that were highly active in the in vitro assay [*Rnf10*, *Nalcn*, *Dnase1/2*(E2), *Dnase1/2*(E7), *Ap4e1*, *Nxn*, *Dbn1*, and *Asic4]* induced the expression of firefly luciferase to levels comparable to the *AAVS1* control; however, the two sgRNAs with less in vitro activity also showed somewhat lower activity in the transfection assay (Fig. 1f). Therefore, these methods provide a simple approach for evaluating the activity of sgRNAs and show that most of the sgRNAs designed from a randomly selected list of genes are active at levels comparable to the highly active *AAVS1* sgRNA.

### CRISPR/Cas9(n) mediated targeting of KOMP constructs in mouse ES cells

**Fig. 2:**
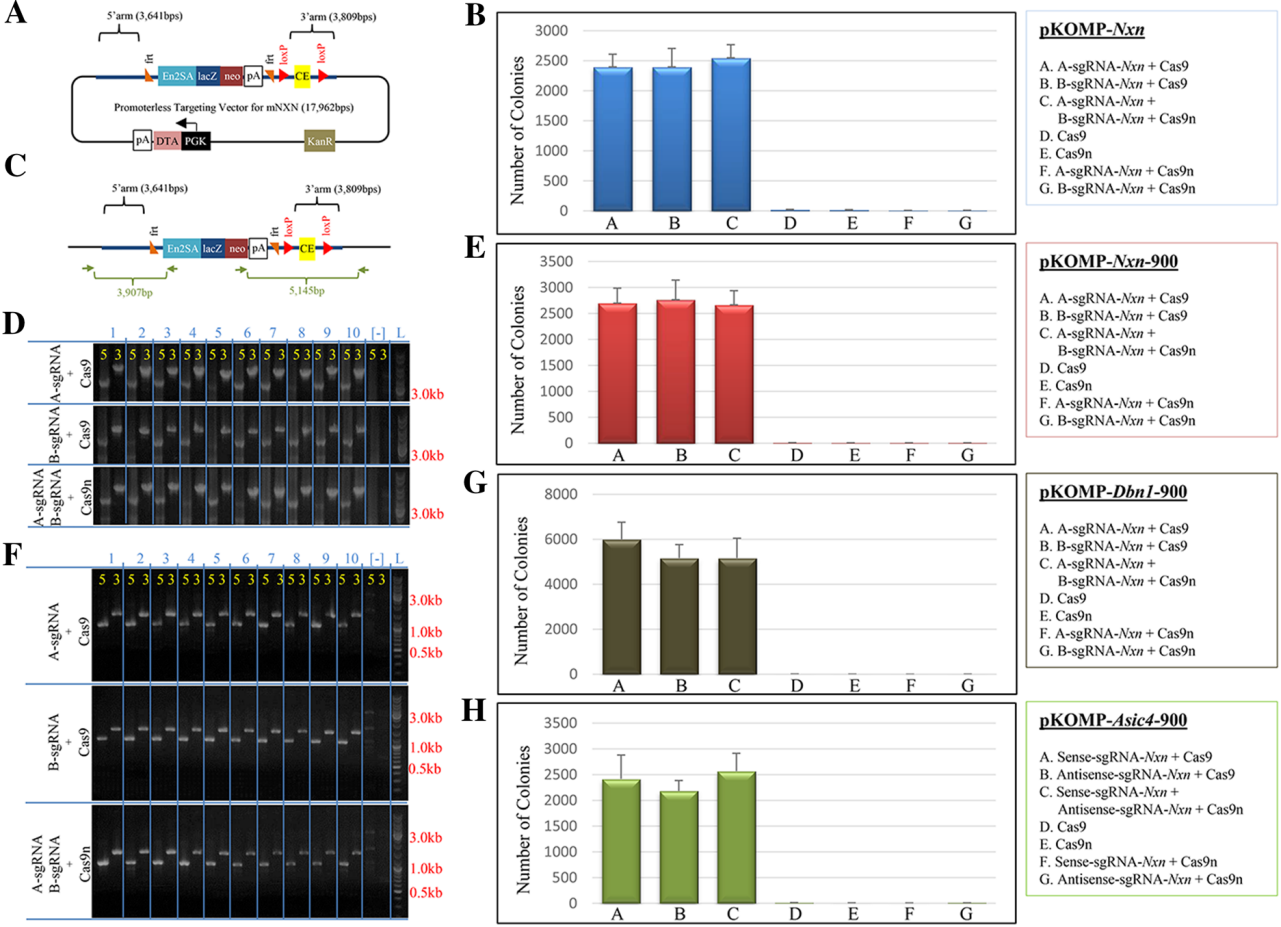
Cas9(n)-mediated targeting efficiency of constructs with long and short homology arms. **a** pKOMP-*Nxn* construct diagram. The promoterless construct was designed such that the lacZ reporter and neomycine/G418-resistance gene were controlled by the endogenous *Nxn* promoter upon proper targeting. The critical exon (CE) was flanked by loxP sequences, therefore, the transgenic mouse derived from this construct can be converted into either a conditional or reporter-marked knockout. **b** *Bar graph* showing the number of G418-resistant ES cell colonies post-electroporation with plasmids expressing sgRNA and Cas9(n), along with the unmodified pKOMP-*Nxn* construct. The results show an increase in the number of colonies in the Cas9(n) mediated conditions (“*A*”, “*B*”, and “*C*”). Conditions with Cas9(n) and pKOMP-*Nxn* without sgRNA(s), and sense- or antisense-sgRNA-*Nxn* with Cas9n and pKOMP-*Nxn,* were used as negative controls (“*D*”, “*E*”, “*F*”, and “*G*”). **c** Diagram illustrating the junction PCR method for verifying correct targeting with products of 3907 bp (5′ arm) and 5145 bp (3′ arm). **d** Junction PCR results for 10 random colonies selected from the G418-resistant colonies. The *top row* shows PCR products from 10 random colonies selected from electroporation using plasmids expressing A-sgRNA-*Nxn* and Cas9, along with pKOMP-*Nxn*. The *middle row* shows B-sgRNA-*Nxn* and Cas9, along with pKOMP-*Nxn*. The *bottom row* shows A-sgRNA-*Nxn*, B-sgRNA-*Nxn* and Cas9n, along with pKOMP-*Nxn*. (−), negative control. **e** Same *bar graph* as (**b**) except using the pKOMP-*Nxn*-900 targeting construct. **f** Same PCR as (**d**) except based for clones generated by the pKOMP-*Nxn*-900 targeting construct. The expected PCR products are 1105 bp (5′ junction) and 1463 (3′ junction). (−), negative control. **g** Same *bar graph* as (**b**) except using the pKOMP-*Dbn1*-900 targeting construct (Fig. 1a). **h** Same *bar graph* as (**b**) except using the pKOMP-*Asic4*-900 targeting construct (Fig. 1a)

Next, we chose one of the genes with sgRNA activity most similar to that of sgRNA-*AAVS1*, *Nxn*, for an in-depth analysis to optimize the parameters for construct targeting stimulated by Cas9(n) in mouse ES cells. The *Nxn* KOMP vector has a multifunctional lacZ reporter and a preconditional “knockout-first” design (Testa et al. 2004; Skarnes et al. 2011) with 3.6 kbp 5′ and 3.8 kbp 3′ homology arms. This design was created for a linearized format with a diphtheria toxin A (DTA) cassette for negative selection to favor homologous targeting over random integration (Fig. 2a). For Cas9-stimulated targeting, the original vector (pKOMP-Nxn, 17.9 kbp) was converted into a derivative that lacks DTA, to use as a circular construct for targeting. The homology arms flank an FRT-encased promoterless lacZ reporter followed by a neomycin/G418-resistant gene that relies on a floxed critical exon and is controlled by an endogenous promoter. To determine whether Cas9(n) enhances targeting efficiency, plasmids expressing A-sgRNA-*Nxn* and Cas9 were co-transfected into mouse ES cells with either pKOMP-*Nxn* or pKOMP-*Nxn*-del that lacked the floxed exon via Cre recombinase (Fig. S3a). Our post-transfection results revealed more than a 2000-fold increase in G418-resistant colonies relative to negative controls, and the PCR analysis of randomly selected colonies showed more than 90% to have constructs correctly targeted in the genome (Fig. 2b–d). The pKOMP-*Nxn*-del vector showed slightly higher targeting success, whereas the pKOMP-*Nxn* vector showed infrequent clones negative for the 3′ loxP PCR, likely resulting from HDR between the 5′ arm and the critical exon. Sequence analysis of the PCR products and copy number analysis using RT-qPCR confirmed the integrity of the targeting (data not shown). We repeated the experiment by co-transfecting plasmids expressing B-sgRNA-*Nxn* with Cas9 or the matched sgRNA-*Nxn* pair with Cas9n, which produced similarly high targeting efficiency (Fig. 2b–d). These observations indicate that sgRNA mediated stimulation of homologous recombination by either a dsDNA break with Cas9 or paired double-nicking with Cas9n enhances pKOMP-Nxn targeting efficiency in mouse ES cells. We also tested for the presence Cas9 plasmid integration in 10 targeted clones and found them to be absent (Fig. S3b).

### Reduction of homology arm length to 900 bp does not effect CRISPR/Cas9(n) mediated targeting efficiency

In designing the KOMP vector (Skarnes et al. 2011), we kept the homology arms larger than 3 kbp to maximize targeting efficiency in mouse ES cells. In light of Cas9(n) increasing the efficiency, we explored how decreasing the length of the homology arms affected targeting. With a single-step gap-repair approach, we shortened the pKOMP-*Nxn* homology arms to 900 bp (pKOMP-Nxn-900). Then we transfected this modified vector into mouse ES cells with plasmids expressing Cas9 and A-sgRNA-*Nxn*; Cas9 and B-sgRNA-*Nxn*; or Cas9n, A-sgRNA-*Nxn,* and B-sgRNA-*Nxn*. Post-transfection, we observed a high number of G418-resistant colonies in all three conditions, similar to transfection with the non-deleted pKOMP-*Nxn* vector (Fig. 2e). Junction PCR analysis of 10 randomly selected colonies from each of the Cas9(n)-mediated conditions confirmed correct targeting in all of the analyzed samples (Fig. 2f). Analysis of different KOMP vectors modified with 900 bp homology arms, pKOMP-*Dbn1*-900 and pKOMP-*Asic4*-900, produced similarly high targeting efficiency (Fig. 2g, h). These observations indicated that Cas9(n) greatly enhances targeting of KOMP vectors in mouse ES cells, and that reducing the homology arm lengths to 900 bp did not compromise the efficiency.

### FokI nuclease fused to catalytically inactive Cas9 decreases targeting efficiency

Recently, Guilinger et al. (2014) and Tsai et al. (2014) used dCas9–FokI in a dimeric form to reduce non-specific genome editing. They reasoned that the obligate dimeric form of dCas9–FokI would cleave the DNA only when two distinct dCas9–Fok1:sgRNA complexes simultaneously bound to adjacent sites with particular spacing constraints (Fig. S4a). While interesting, whether dCas9–FokI can induce HDR for efficient construct targeting remains unknown. Specifically, the stringent spatial requirements for assembling dCas9–FokI dimers and the bulky hybrid protein may affect the efficiency of construct targeting.

We sought to compare the targeting efficiencies of pKOMP-*Nxn*-900 mediated by Cas9n (Fig. S4b) and dCas9–FokI (Fig. S4a). We designed a new FokI-sgRNA-*Nxn* as an obligate dimer with the A-sgRNA-*Nxn*, such that a 24 bp spacer region separated the two sgRNA-binding sequences (Fig. S4a); the 24 bp distance was determined based on a report by Guilinger et al. (2014) in which a ~15 or ~25 bp spacer distance between the dimeric sgRNA:dCas9–FokI was optimal for gene modification. Our findings show that co-transfecting four plasmids expressing A-sgRNA-*Nxn*, FokI-sgRNA-*Nxn,* and Cas9n, along with pKOMP-*Nxn*-900, reduced the number of G418-resistant colonies to approximately half of those observed in conditions where B-sgRNA-*Nxn* was used instead of FokI-sgRNA-*Nxn* (Fig. S4c). When the same transfection conditions were repeated to replace Cas9n with dCas9–FokI, the number of G418-resistant colonies reduced to a similar level as the negative controls (Fig. S4c). These observations suggested that while obligate dimeric dCas9–FokI may reduce off-target activities of sgRNAs, the low efficiency of construct targeting must be improved.

### Homology arm length and symmetry are critical for Cas9(n) mediated construct targeting

**Fig. 3:**
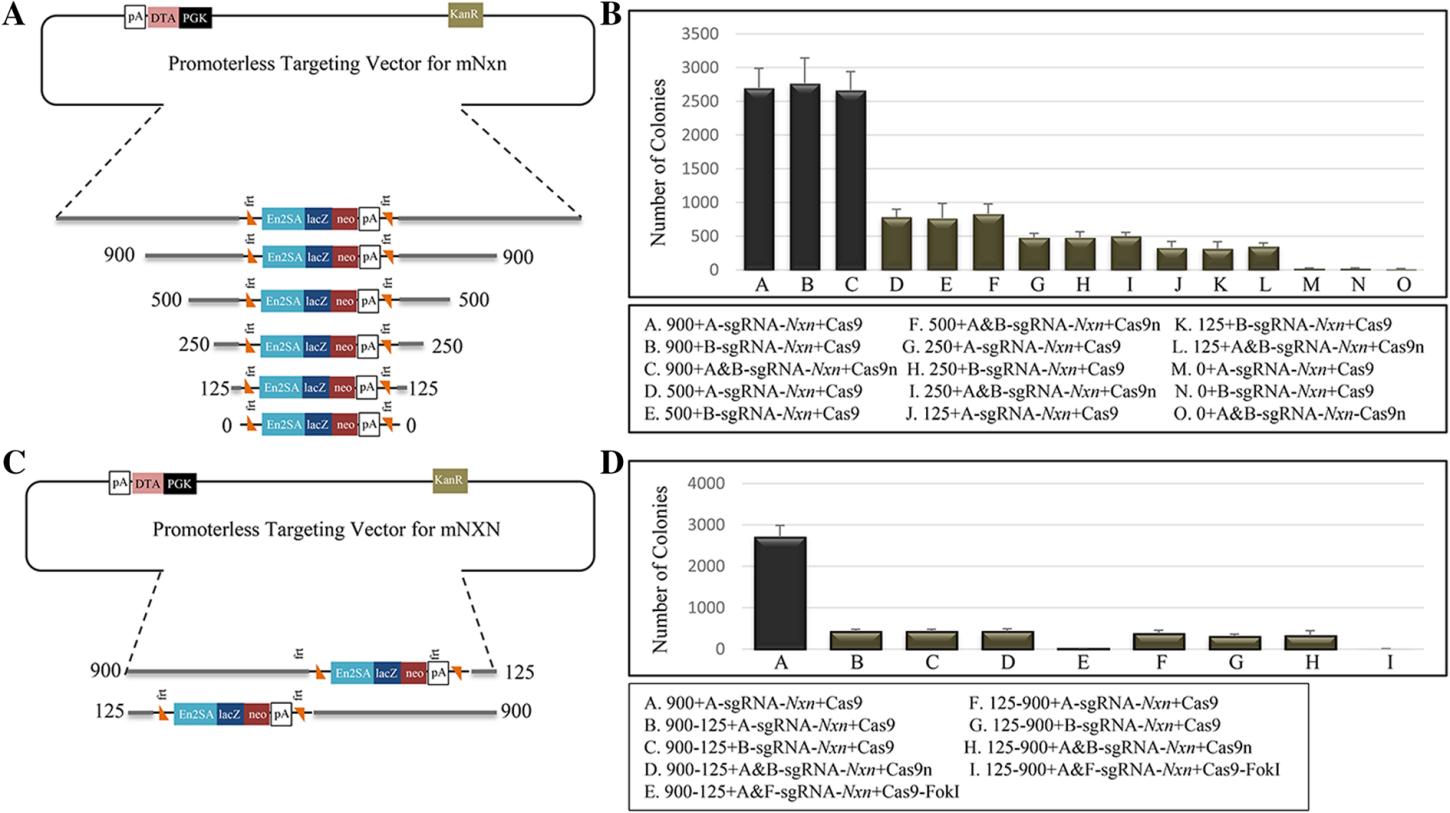
Homology arm length and symmetry influence targeting efficiency. **a** Diagram illustrating pKOMP-*Nxn* constructs with various symmetrical homology arm lengths. **b** *Bar graph* showing the number of G418-resistant colonies post-electroporation with pKOMP-*Nxn* with five different homology arm lengths (900, 500, 250, 125, and 0 bps). **c** Diagram illustrating pKOMP-*Nxn* constructs with asymmetrical homology arm lengths. **d** *Bar graph* showing the number of G418-resistant colonies post-electroporation with pKOMP-*Nxn* carrying asymmetrical homology arm lengths. F-sgRNA-*Nxn* = FokI-sgRNA-*Nxn*

Next, we wondered whether we could further decrease the homology arm length without ramifications. We reduced the homology arm lengths of pKOMP-*Nxn* to 500, 250, 120, and 0 bp (Fig. 3a) and transfected the modified targeting constructs with plasmids expressing Cas9 and A-sgRNA-*Nxn*; Cas9 and B-sgRNA-*Nxn*; and Cas9n, A-sgRNA-*Nxn,* and B-sgRNA-*Nxn*. We observed about a four-fold drop in the number of colonies upon shortening from 900 bp to 500 bp and further proportional decreases with shorter arm lengths of 259 and 125 bp (Fig. 3b). Because these findings indicated that the homology arm lengths must be close to 900 bp to maintain high targeting efficiency, we examined whether a single 900 bp arm would suffice. We thus modified the pKOMP-*Nxn* vector to have a 900 bp homology arm at one end and a 125 bp arm at the other end (Fig. 3c). We transfected these constructs with plasmids expressing Cas9, Cas9n, or dCas9–FokI, along with the corresponding sgRNAs, which revealed that modifying the targeting constructs with asymmetric homology arms drastically decreased the number of G418-resistant colonies to a level comparable to constructs having both arm lengths at 125 bp. Thus, constructs with dual homology arms of at least 900 bp must be used to obtain high targeting efficiency.

### Cas9 protein yields higher targeting efficiency in mouse zygotes than Cas9 mRNA

Next, we focused on construct targeting in mouse zygotes with the same approach used in ES cell transfection. We selected one of the KOMP vectors used in previous ES cell transfection experiments (pKOMP-*Asic4*-900) for targeting in zygotes. In an initial experiment, we co-injected the pKOMP-*Asic4*-900 vector with either Cas9 protein or mRNA and A-sgRNA-*Asic4*. Injections with Cas9 protein (Table 1) produced one out of two founder pups with targeted *Asic4* integration (50%), while injections with Cas9 mRNA produced zero out of four founder pups (0%) targeted. This experiment was repeated using a construct with longer (2 kbp) homology arms, which produced 6 out of 17 pups with targeted integration for the Cas9 protein (35%), as compared to 1 out of 28 for Cas9 mRNA (4%). Random selection of one of the founder pups for mating showed that the transgenes were germline transmissible, segregating according to Mendelian genetics (8 of 12 F1 pups were heterozygotes) (Fig. S5). These findings indicate that Cas9 protein is more efficient than Cas9 mRNA at stimulating HDR, thereby increasing the rate of construct targeting via homologous recombination.

**Table 1:** CRISPR Cas9 mRNA versus Cas9 protein mediated targeting of KOMP construct in mouse zygotes

Targeting vector ID	Cas9 mRNA versus protein	Cas9 (ng/μl)	sgRNA (ng/μl)	Targeting vector (ng/μl)	# Embryos transferred	# Of pups	Genotype positive	% Positive relative to # of embryos transferred	% Positive relative to # of pups
pKOMP-*Asic4*	mRNA	20	10	20	81	4	0	0	0
(900 bp HAs)	Protein	50	25	20	52	2	1	2	50
pKOMP-*Asic4*	mRNA	20	10	10	78	28	1	1	4
(2000 bp HAs)	Protein	50	25	10	51	17	6	12	35

### Strategy for CRISPR/Cas9 mediated deletion of critical exons and multi-vector targeting through a single zygote injection experiment

Thus far, we have shown that sgRNA:Cas9(n) complexes designed to induce DNA cleavage in the critical exon sequence stimulate highly efficient targeting of KOMP vectors with symmetrical homology arms as short as 900 bp. In this strategy, additional mutant alleles with disrupted gene function may result from insertions/deletions (indels) in the coding sequence generated by imprecise break repair by the NHEJ pathway (Ran et al. 2013). However, NHEJ events are unpredictable and may produce unexpected splicing and/or frame shifts. Thus, we shifted our strategy toward creating dual breaks that flank coding exons, such that alleles lacking construct integration may, instead, be subject to critical exon deletion via NHEJ, resulting in two types of easily characterized mutations.

Recently, Zhou et al. (2014) showed that chromosomal DNA larger than 100 kbp can be deleted using multiple sgRNA:Cas9 complexes flanking the targeting DNA segment. Thus, we aimed to evaluate whether a pair of sgRNAs targeting regions flanking the critical exon could knock-in the conditional construct through homologous recombination, or alternatively, to delete the entire critical exon. To test the viability of this strategy, we designed two sgRNAs that target regions flanking the critical exon of the *Nxn* gene. We planned to use these constructs with pKOMP-*Nxn*-900, such that the guide RNA target sequences were eliminated by either targeted construct integration or a deletion between the up- and downstream cleavage sites (Fig. S6a) (hereafter referred to as Upstream-sgRNA-*Nxn*-CE and Downstream-sgRNA-*Nxn*-CE). The dsDNA cleavage efficiencies of the sgRNAs were validated using the SSA firefly luciferase assay (Fig. 1e) to ensure that their activities were comparable to that of sgRNA-*AAVS1*, A-sgRNA-*Nxn,* and B-sgRNA-*Nxn* (Fig. S6b). We then co-transfected plasmids expressing Upstream-sgRNA-*Nxn*-CE, Downstream-sgRNA-*Nxn*-CE, and Cas9, along with pKOMP-*Nxn*-900, which revealed that the number of G418-resistant colonies was similar to that of A-sgRNA-*Nxn* and Cas9 and A-sgRNA-*Nxn*, B-sgRNA-*Nxn,* and Cas9n (Fig. S6c). Junction PCR results of 10 random colonies confirmed correct targeting of eight colonies, on average, that were positive at both homology arm junctions, and one or two colonies that were only positive at the 5′ homology arm (data not shown). The positive junction PCR at just one of the homology arms may have resulted from a recombination event occurring at the critical exon sequence instead of at the 3′ homology arm. Hence, while using a pair of sgRNAs flanking the critical exon slightly reduced targeting efficiency for pKOMP-*Nxn*-900, the efficiency was still at least 2000-fold higher relative to the negative control (Fig. S6c).

**Fig. 4:**
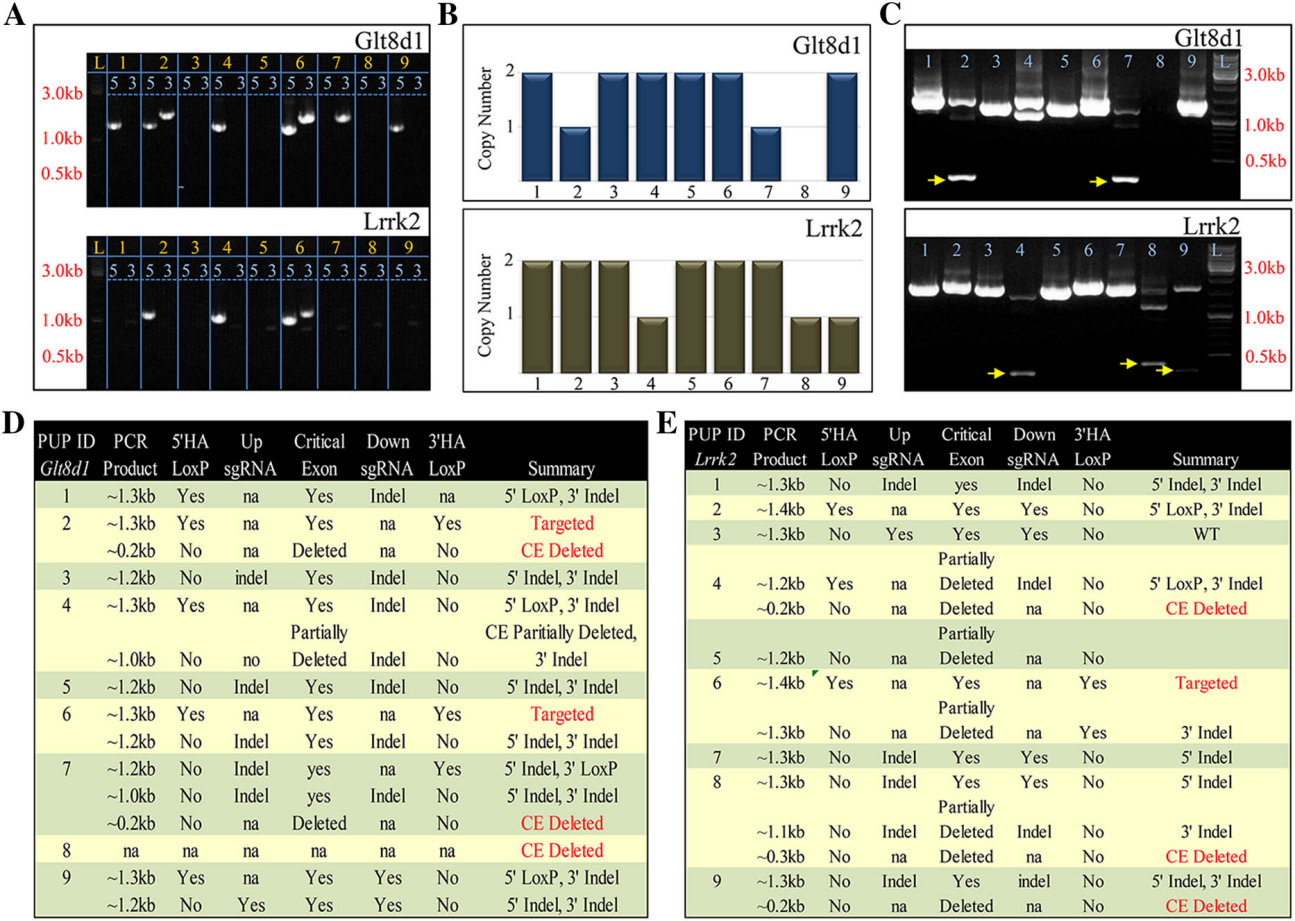
CRISPR/Cas9 induces efficient multi-vector targeting via HDR and NHEJ mediated gene knockout via a single injection to mouse zygotes. **a** Junction PCR at 5′ and 3′ homology arms to verify targeting of pKOMP-*Glt8d1* and pKOMP-*Lrrk2* in the nine founder pups. **b** RT-qPCR to evaluate copy number of *Glt8d1* and *Lrrk2* critical exons in the nine founder pups. **c** PCR to determine critical exon deletion due to sgRNAs targeting regions flanking the critical exons of *Glt8d1* or *Lrrk2* in the nine founder pups.* Yellow arrows*, amplicons resulting from critical exon deletions. **d**, **e** Summary of sequencing data derived from the gel-extracted PCR products from (**c**). *First columns*, ID of pups for *Glt8d1* and *Lrrk2*; *second columns*, approximate size of the PCR products that were gel extracted from (**c**); *third columns*, indicate presence of 5′HA loxP sequences; *fourth columns*, indicate presence of indels in the Upstream-sgRNA target regions—not applicable (na), indicates presence of 5′HA LoxP; *fifth columns* indicate it the critical exons are present, deleted, or partially deleted; *sixth columns*, show presence of indels in the Downstream-sgRNA target regions—not applicable (na) indicates presence of 3′HA LoxP; *seventh columns* indicates presence of 3′HA loxP sequences; *last column*, summary of sequence data. *Red letters* indicate targeted or critical exon (CE)—deleted alleles. (Color figure online)

After observing that a pair of sgRNAs flanking a critical exon maintains high targeting efficiency in ES cells, we hypothesized that this high targeting efficiency would allow targeting of multiple genes by performing a single injection into zygotes that contains corresponding constructs. We also speculated that by using two sgRNAs flanking critical exons, we could obtain additional null alleles by deleting entire exons via NHEJ. To this end, we used two KOMP vectors with floxed exon regions (pKOMP-*Lrrk2*-900 and pKOMP-*Glt8d1*-900) and sgRNAs targeting genomic sequences not present in the constructs (Fig. S7a, b). We co-injected both targeting constructs, along with two pairs of sgRNA transcripts and Cas9 protein, in mouse zygotes, which produced nine founder pups. Among them, two of the pups contained a floxed *Lrrk2* allele (22%); one of these pups also had a floxed *Gltd81* allele (11%) (Fig. 4a; Table 2). Copy number analysis by RT-qPCR of the *Lrrk2* and *Glt8d1* regions in nine of the pups revealed that two of them (22%) had single-exon deletions for *Lrrk2*, three (33%) had single-exon deletions for the *Glt8d1*critical region, and one (#8) had a homozygous exon deletion for *Glt8d1* (Fig. 4b). We further characterized the deletion alleles by diagnostic PCR spanning the critical exon (Fig. 4c). Not surprisingly, pups with a single-exon deletion by RT-qPCR showed diagnostic PCR bands that confirmed the predicted deletion allele. The absence of any *Glt8d1* PCR products in pup #8 suggested that the deletion may include primer-binding sites. Surprisingly, *Glt8d1* pup #2 was heterozygous pKOMP-*Glt8d1*-900, combining the floxed and deletion allele in a single founder. Next, we gel extracted the visible PCR bands, inserted them into a cloning vector, and randomly picked two or three colonies for sequencing. Sequence analysis of *Glt8d1* and *Lrrk2* (Fig. 4d, e) showed that only one of the nine pups (#3) was wild type for both *Lrrk2* alleles. As such, the overwhelming majority of the targeted alleles had proper construct targeting, had complete or partial deletion of critical exons, and/or obtained indels at the sgRNA target regions. These findings support that using a pair of sgRNAs flanking the critical exon is a highly efficient method for targeting multiple constructs in a single zygote injection experiment, as well as for inducing NHEJ mediated deletion of the critical exon for knocking out gene function in non-targeted alleles.

**Table 2:** CRISPR/Cas9 protein mediated multi-vector targeting in mouse zygotes

Targeting vector ID	Cas9 protein (ng/μl)	sgRNA (ng/μl)	Targeting vector (ng/μl)	# Embryos transferred	# Pups	Genotype positive *Glt8d1*	Genotype positive *Lrrk2*	Genotype positive *Gltsd1* and *Lrrk2*	% Positive relative to # embryos transferred *Glt8d1*	% Positive relative to # embryos transferred *Lrrk2*	% Positive relative to # embryos transferred *Glt8d1* and *Lrrk2*	% Positive relative to # pups *Glt8d1*	% Positive relative to # pups *Lrrk2*	% Positive relative to # pups *Glt8d1* and *Lrrk2*
pKOMP-*Glt8d1*-														
(~900 bp HAs)		10 + 10	5											
&	50	&	&	62	9	1	0	1	2	0	2	11	0	11
pKOMP-*Lrrk2*-														
(~900 bp HAs)		10 + 10	5											

## Discussion

In this study, we showed that targeting constructs in mouse zygotes with high efficiency is possible through the HDR pathway mediated by the CRISPR/Cas9 system. With a systematic approach, we examined the dsDNA cleavage activity of sgRNAs, determined the role of homology arm length in the targeting constructs, and compared the efficiencies of Cas9, Cas9nickase, and dCas9–FokI in an ES cell model. This approach revealed three interesting findings. First, most of the sgRNA evaluated were highly active. Second, decreasing the homology arm length from approximately 3 kbp to 900 bp did not affect targeting efficiency; however, further decreasing the length and disturbing the symmetry between the two arms considerably decreased the efficiency. Third, while the sgRNA complexed with either Cas9 or Cas9n gave rise to high targeting, switching the endonuclease to dCas9–FokI dramatically lowered the efficiency to a level comparable to the background. After we established the optimal conditions in the ES cell model, we translated them into zygotes with either Cas9 mRNA or protein to further hone and improve the targeting parameters. Similar to our observations in ES cells, we found that the targeting efficiencies in zygotes were comparable between constructs harboring 900 bp or 2 kbp homology arm lengths. Interestingly, we observed higher targeting efficiencies when Cas9 protein was used instead of mRNA, similar to that observed by Menoret and colleagues in rat and mouse zygotes (Menoret et al. 2015).

The successful outcome using constructs with 900 bp homology arms and Cas9 protein in zygotes led us to wonder whether we could target multiple vectors in a zygote with a single injection. Here, we designed a new strategy for construct targeting of two different genes for conditional knockout via HDR and deletion of gene function via NHEJ by introducing two pairs of sgRNAs flanking critical exons in a single injection to a zygote. This strategy provided a proof of concept, and for the first time, demonstrated that multiple genes could be targeted for conditional knockout through the HDR pathway and for direct knockout of their functions in non-targeted alleles through a single injection to a zygote. Our findings demonstrate that by systematically optimizing conditions, we established an efficient, robust, and reliable method for construct targeting in zygotes mediated by CRISPR/Cas9.

While developing this study, concerns were raised regarding the possibility that a large fraction of the sgRNAs may possess low cleavage activity, likely requiring each sgRNA to be carefully tested to develop an optimal procedure for construct targeting. Thus, we designed two different assay systems (i.e., the in vitro Cas9 protein assay and the ex vivo SSA assay) for evaluating DNA-cleavage activity. In using these assays, however, we discovered that most of the sgRNAs were highly active at levels comparable to the sgRNA-*AAVS1* reported by Mali and colleagues (2013). Based on this result, we speculated that the optimization procedure should shift its focus from evaluating sgRNA activity to minimizing the length of the homology arms to decrease the size of the constructs and to identifying the most efficient type and form of Cas9 endonucleases. While evaluating the endonucleases, we explored the option of using the obligate dimeric properties of dCas9–FokI to strengthen sgRNA specificity, because some reports raised concerns about high off-target mutation rates induced by CRISPR/Cas9 in human cells (Fu et al. 2013; Hsu et al. 2013; Pattanayak et al. 2013; Lin et al. 2014). Unfortunately, experiments using sgRNA complexed with dCas9–FokI yielded targeting efficiencies comparable to that of background. Many reports suggest that off-target activities may depend on sgRNA target sequences, and that the off-target activities of more promiscuous sgRNAs can be kept at a minimum by engineering them with extra guanines at the 5′ terminus or by choosing unique target sequences near the PAM distal region. This engineering would avoid target sequences with one or two mismatches in other genomic loci (Yang et al. 2013; Cho et al. 2014; Smith et al. 2014; Veres et al. 2014; Kim et al. 2015). Thus, designing sgRNAs with unique target sequences may sufficiently minimize potential off-target effects in animal models.

In addition to a reliable and robust strategy to minimize potential off-target effects, we also need to examine whether conditions described in this study can successfully target more than two constructs in a single injection to a zygote, and whether larger constructs, such as bacterial artificial chromosomes (BACs), can be targeted without losing efficiency. Recently, Yoshimi et al. (2016) described a procedure in which they injected rat zygotes with poly(A) elongated Cas9 mRNA and two sgRNAs and 80 bp ssODNs overlapping the DNA cleavage sites to knockin a ~200 kbp BAC (human SIRPa). With this method, they successfully targeted 1 of 15 founder pups. While they also report that this strategy has the major disadvantage of a high rate of indel mutations at the ssODN mediated conjunction sites, they did not determine whether the strategy could be successfully and reliably reproduced in rat and mouse zygotes.

In summary, we have described an optimized condition for CRISPR/Cas9-mediated construct targeting in mouse zygotes. Our study adds to the growing body of literature describing a myriad of new technological advancements, and, together, they enhance our ability to manipulate the genome. Ultimately, these tools are an essential part of biological sciences, and they facilitate biomedical and translational research toward improving human health.

## Experimental procedures

### sgRNA design, expression vectors, and transcription

#### Design

sgRNAs were designed using the CRISPR guide–design tool developed by the Zhang laboratory at MIT (crispr.mit.edu).

#### Expression vectors

sgRNA sequences were cloned into U6 target gRNA expression vector as described by Mali et al. (2013).

#### Transcription

sgRNA templates were amplified with T7-promoter-sequence conjugated primers and purified using a PCR cleanup kit (Machery-Nagel). Amplified products were used as templates for transcription using the MEGAshortscript T7 Kit (Thermo Fisher Scientific). Transcripts were purified using the MEGAclear Kit (Thermo Fisher Scientific).

### SSA and in vitro cleavage assay

#### SSA assay

The SSA assay was performed as previously described (Ochiai et al. 2010). Briefly, the target sequence of each sgRNA was cloned into the pGL4-SSA reporter vector and co-tranfected into HEK293T cells with pRL-CMV (Promega) and sgRNA-expressing plasmid. Twenty-four hours post-transfection, firefly and renilla luciferase quantification was done using the Dual-Glo Luciferase Assay Kit (Promega) following the manufacturer’s instructions.

#### In vitro cleavage assay

The pGL4-SSA plasmid carrying the sgRNA-target sequence (200 ng) was incubated with Cas9 protein (500 ng; PNA Bio) and sgRNA transcripts (50 ng) at 37 °C for 1 h, followed by heat inactivation at 65 °C for 10 min and proteinase K treatment for 30 min at 60 °C.

### KOMP vector modifications

To modify the homology arm length of KOMP vectors, a two-step approach was used. First, the vector inserts were dissociated from the vector backbone using PacI and AsiSI and cloned into a low copy—vector backbone with a different antibiotic-resistance marker using Gibson Assembly (NEB). Then, the inserts were gap-repaired into the pUC19 vector backbone with various homology arm lengths.

### Cell culture and transfection

#### ES cell culture

Mouse JM8.F6 embryonic stem (ES) cells were obtained from the Mouse Biology Program at the University of California, Davis. Cells were maintained as a monolayer on 6-well (9.6 cm^2^) plates on feeder layers of γ-irradiated mouse embryonic fibroblasts (MEF) (Global Stem) in Dulbecco’s Modified Eagle Medium (DMEM; Thermo Fisher Scientific) supplemented with 15% fetal bovine serum (FBS; Hyclone), 1000 U/ml leukemia-inhibitory factor (Millipore), 1 mmol/l non-essential amino acids (Thermo Fisher Scientific), 2 ml l-glutamine (Thermo Fisher Scientific), and 0.01 mmol/l 2-mercaptoethanol (Thermo Fisher Scientific).

#### Electroporation and G418 selection

On the day of electroporation, ES cells were trypsinized, separated into single cells, and placed in a 37 °C incubator for 1 h as a suspension culture in 100 mm plates coated with 2% gelatin. Then, 10^7^ cells were electroporated using BTX (700 V, 400 Ω, 25 μF) with 15 μg Cas9(n) and 15 μg sgRNA(s)-expressing plasmids, along with 15 μg of the targeting vector. Electroporated cells were placed on 6-well plates with a monolayer of γ-irradiated DR4 MEF (Global Stem) feeders. Two days post-transfection, medium was supplemented with 150 μg/ml G418, and the selection continued for 7–10 days.

### Genomic DNA isolation and genotyping

#### Genomic DNA isolation

gDNA was isolated from tail biopsies by adding 500 μl of lysis buffer (10 mM Tris, 100 mM NaCl, 10 mM EDTA, 0.5% SDS) and 20 μl of proteinase K (20 mg/ml) and incubating them overnight in a 60 °C water bath. Then, 250 μl of 6 M NaCl was added to each tube, which was centrifuged at 8000 rpm for 10 min before the supernatant was transferred to new tubes. Isopropanol was added to precipitate the DNA, and 70% ethanol was used to wash the pellet.

#### Genotyping

Genotyping was done using Sequal Prep Long PCR Kit (Thermo Fisher Scientific). Here, 20 ng of genomic DNA was used as template and amplified following the manufacturer’s instructions. All primer sequences are listed in Figure S8.

### Real-time quantitative PCR (qPCR)

Using 20 ng of isolated genomic DNA, qRT-PCR reactions were performed with SYBR Green PCR Master Mix (Applied Biosystems) according to the manufacturer’s instructions. Here, 200 nM of each primer pair were used to detect critical exons of *Glt8d1* or *Lrrk2*; *Gapdh* served as the relative control. Ct values were calculated using Applied Biosystems’ SDS2.4 software, and the Ct values derived from *Glt8d1* or *Lrrk2* were normalized to the *Gapdh* gene in the mouse genome to determine the copy number.

### Mouse zygote injections

All animal procedures were approved by the Institutional Animal Care and Use Committee at University of California, San Francisco. Super-ovulated female FVB/N mice (4-weeks-old) were mated to FVB/N stud males, and fertilized zygotes were collected from oviducts. Cas9, sgRNA, and plasmid vectors were mixed and injected into the pronucleus of fertilized zygotes. The concentrations of Cas9 protein, Cas9 mRNA, sgRNAs, and plasmid vectors are described in Tables 1 and 2. After the injection procedure, zygotes were implanted into oviducts of pseudopregnant CD1 female mice.

## Electronic supplementary material

Below is the link to the electronic supplementary material.

Supplementary material 1 (PDF 672 kb)
