# Supplementary material for: Efficient gene targeting in mouse zygotes mediated by CRISPR/Cas9-protein
Source: Transgenic Res. 2016 Nov 30;26(2):263–77. doi: 10.1007/s11248-016-9998-5 (PMC5350237; doi:10.1007/s11248-016-9998-5)
Supplement: Supplementary file 1 — Supplementary material 1 (PDF 672 kb) [file 11248_2016_9998_MOESM1_ESM.pdf]

Supplemental Figure 1

A

Gene ID	MGI	Chr
Ano3	3613666	2
Rnf10	1859162	5
Nalcn	2444306	14
Dnase1/2	1913955	17
Ap4e1	1336993	2
Ces1d	2148202	8
Nxn	109331	11
Dbn1	193183	13
Asic4	2652846	1

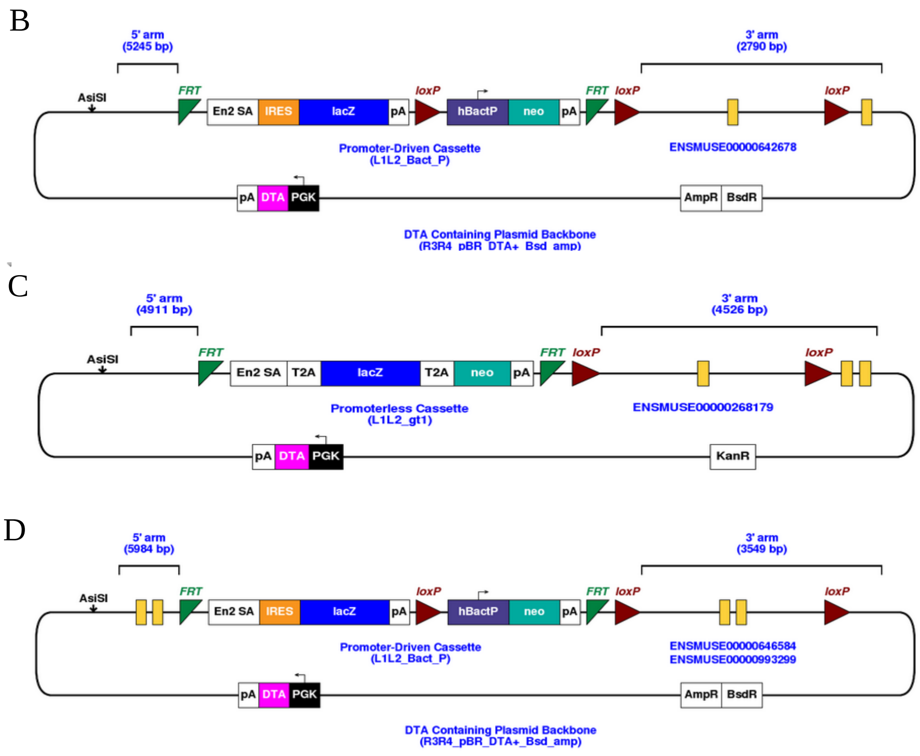

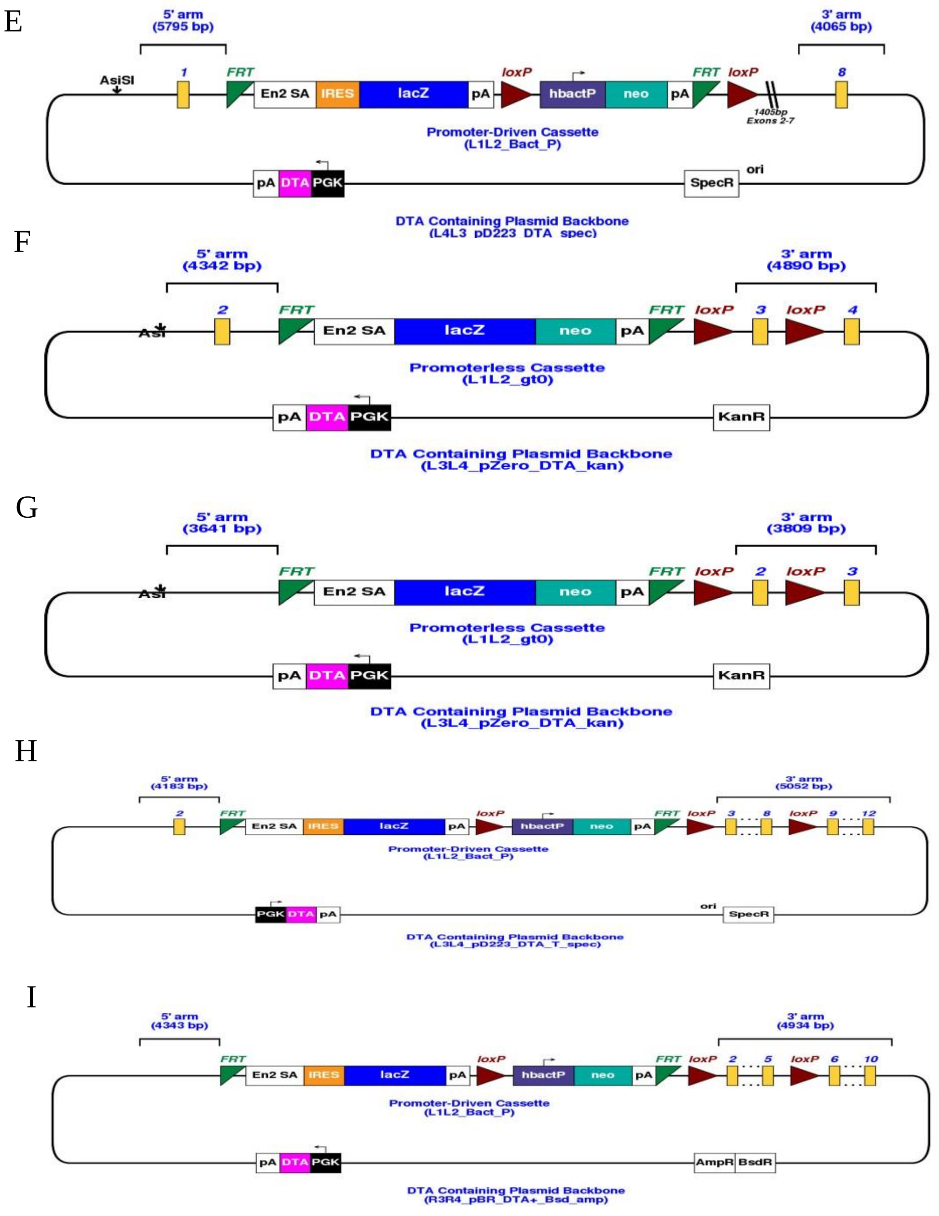

Supplemental Figure 1. Selected KOMP Constructs. (A) Table listing nine KOMP vectors randomly selected from the BACPAC KOMP Vector Repository. Left column, gene I.D.; middle column, Mouse Genome Informatics (MGI) I.D.; right column, chromosome location. Illustrations of selected KOMP constructs for (B) Ano3 (C) Rnf10 (D) Nalcn (E) Dnase1/2 (F) AP4e1 (G) Nxn (H) Dbn1 (I) Asic4.

Supplemental Figure 2

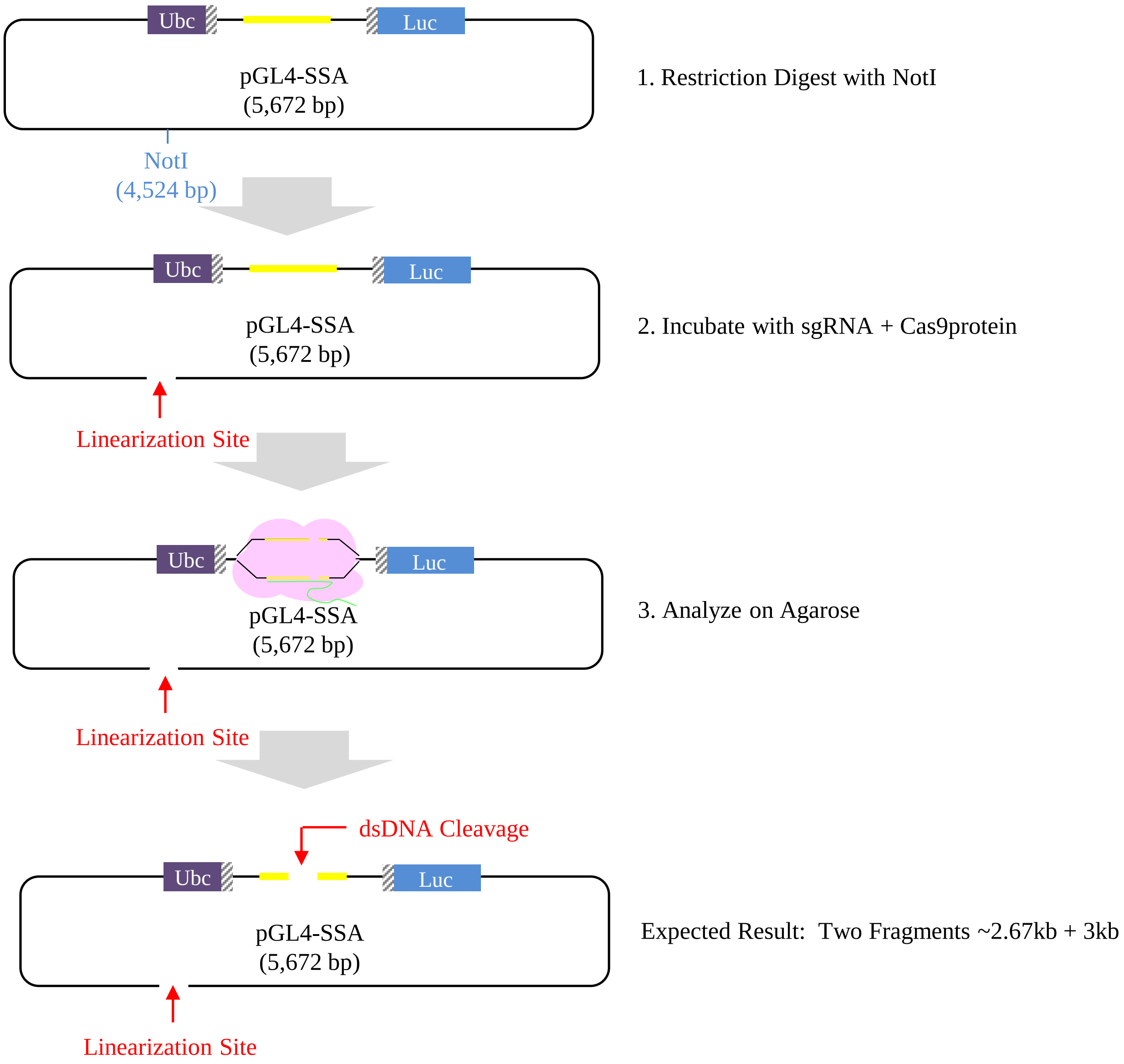

Supplemental Figure 2. ***In Vitro* Cas9 Protein Assay.** Schematic diagram illustrating the SSA assay. The pGL4-SSA that has been cloned with sgRNA target sequence is linearized with NotI. The linearized vector is incubated with sgRNA transcript and Cas9protein, and analyzed on agarose gel. If the dsDNA cleavage was induced by the sgRNA:Cas9protein complex, two bands are expected, size range ~2.67kb and 3kb.

Supplemental Figure 3

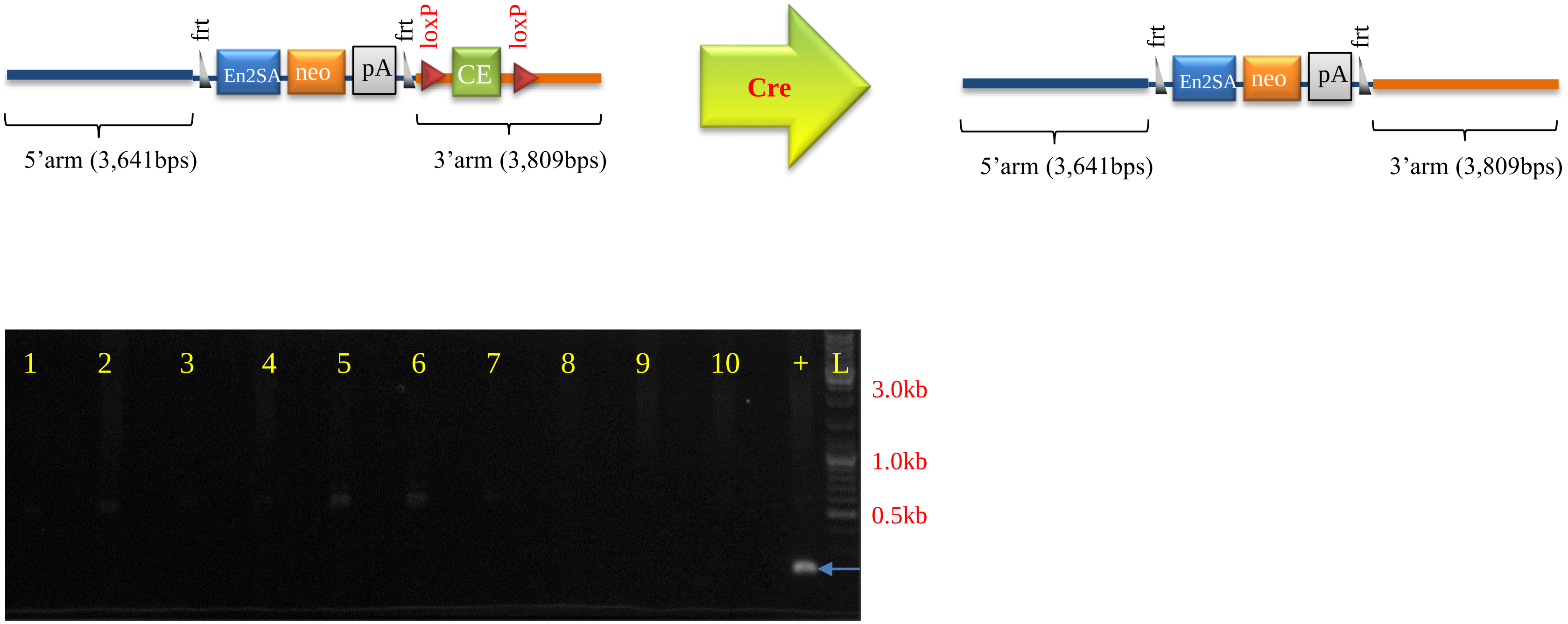

Supplemental Figure 3. Cre Removal Strategy & Analysis of Cas9 Plasmid Integration. (A) Diagram illustrating the removal of the critical exon using Cre recombinase. (B) PCR analysis showing absence of Cas9 expressing plasmid in targeted ES cell clones. Numbers show random ES cells clones that were targeted. + shows positive control using wildtype gDNA with 0.0001pg Cas9 plasmid. L shows marker lane. Blue arrow shows PCR band positive for the presence of Cas9 plasmid.

Supplemental Figure 4

A

B

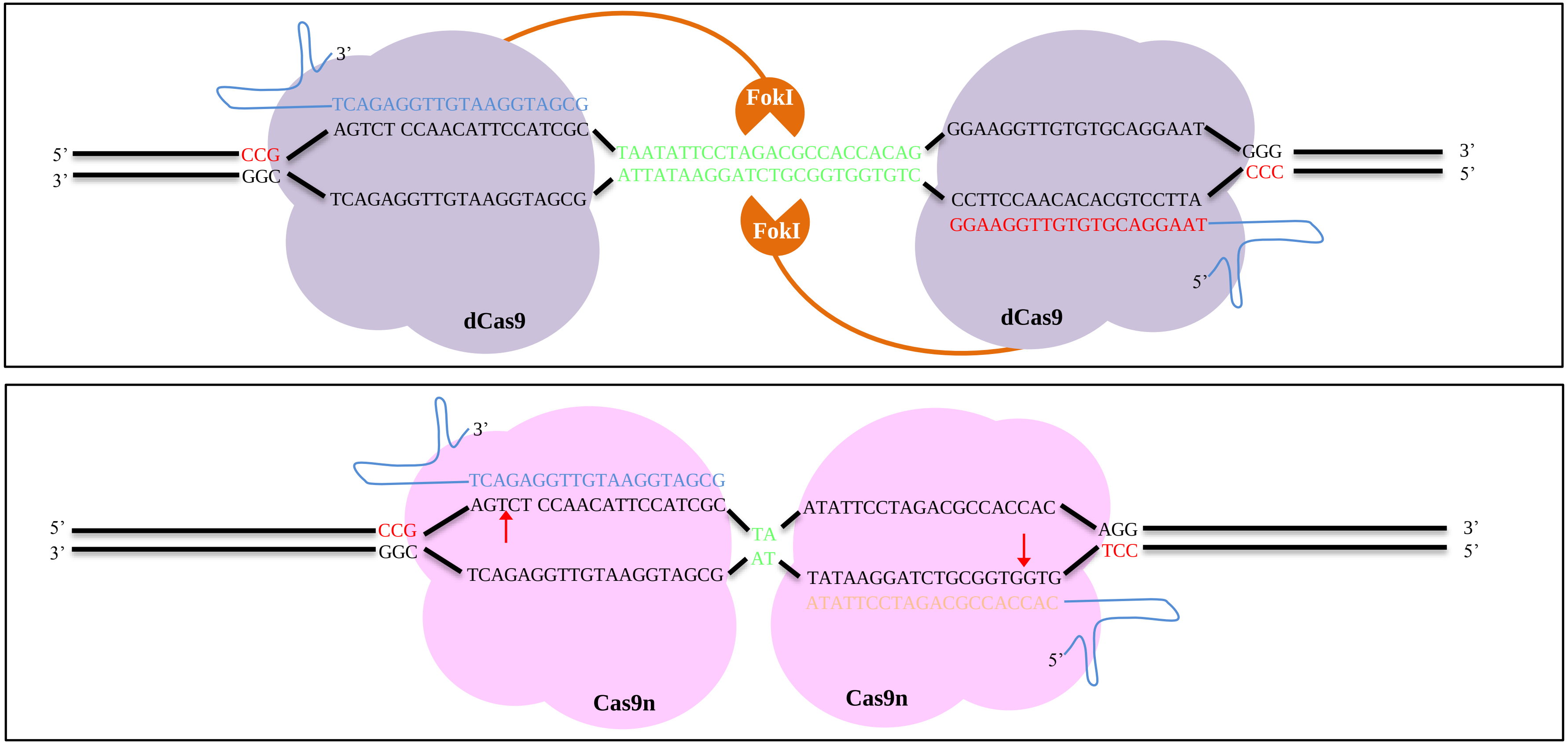

C

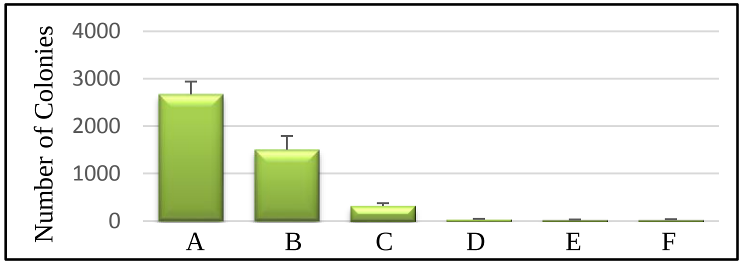

A. A-sgRNA-*Nxn* + B-sgRNA-*Nxn* + Cas9n
B. A-sgRNA-*Nxn* + FokI-sgRNA-*Nxn* + Cas9n
C. A-sgRNA-*Nxn* + FokI-sgRNA-*Nxn* + dCas9-FokI
D. B-sgRNA-*Nxn* + FokI-sgRNA-*Nxn* + dCas9-FokI
E. Cas9n
F. dCas9-FokI

Supplemental Figure 4. Comparative Analysis of Construct Targeting Efficiency Between Cas9d and dCas9-FokI. (A) Diagram illustrating the mechanistic architecture of obligate dimeric dCas9-FokI. The FokI endonuclease is fused to catalytically inactive Cas9. The A-sgRNA-*Nxn* (blue) complexed with dCas9 binds to the upstream segment of the target *Nxn* critical exon. The FokI-sgRNA-*Nxn* (red) complexed with dCas9-FokI binds to the opposite DNA strand 24 bp downstream. Double strand DNA cleavage is induced by FokI only when the up- and downstream sgRNA:dCas9-FokI complexes form a dimer. (B) Diagram illustrating the details of sgRNA:Cas9n complexes. The blue sgRNA represent A-sgRNA-*Nxn*, and the orange sgRNA represent B-sgRNA-*Nxn* (**Fig. 1b**). Red arrows indicate site where nicks are induced. (C) G418 resistant colony numbers post ES cell electroporation with plasmids expressing either Cas9n or dCas-FokI, along with sgRNAs and pKOMP-*Nxn*-900.

Supplemental Figure 5

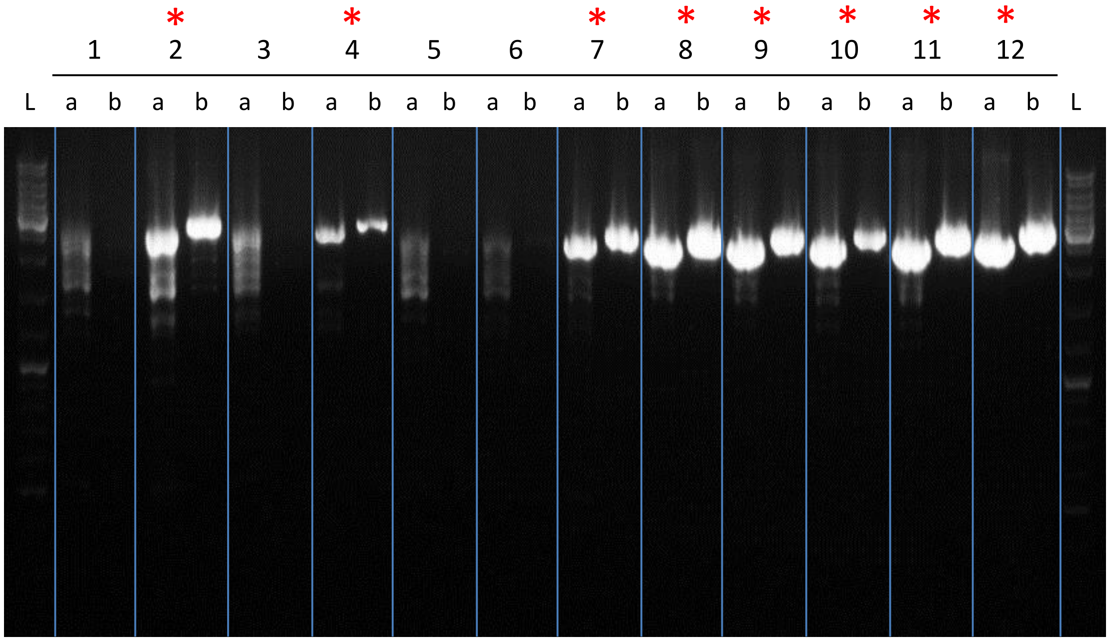

a: 5’ homology arm junction
b: 3’ homology arm junction
L: Ladder
*: Both junctions positive

Supplemental Figure 5. Genotyping Result for F1 pKOMP-Asic4-900. Genotyping result showing F1 pups derived from one of the pKOMP-Asic4-900 zygote injection experiment using Cas9protein. 8 of 12 pups are genotype positive.

Supplemental Figure 6

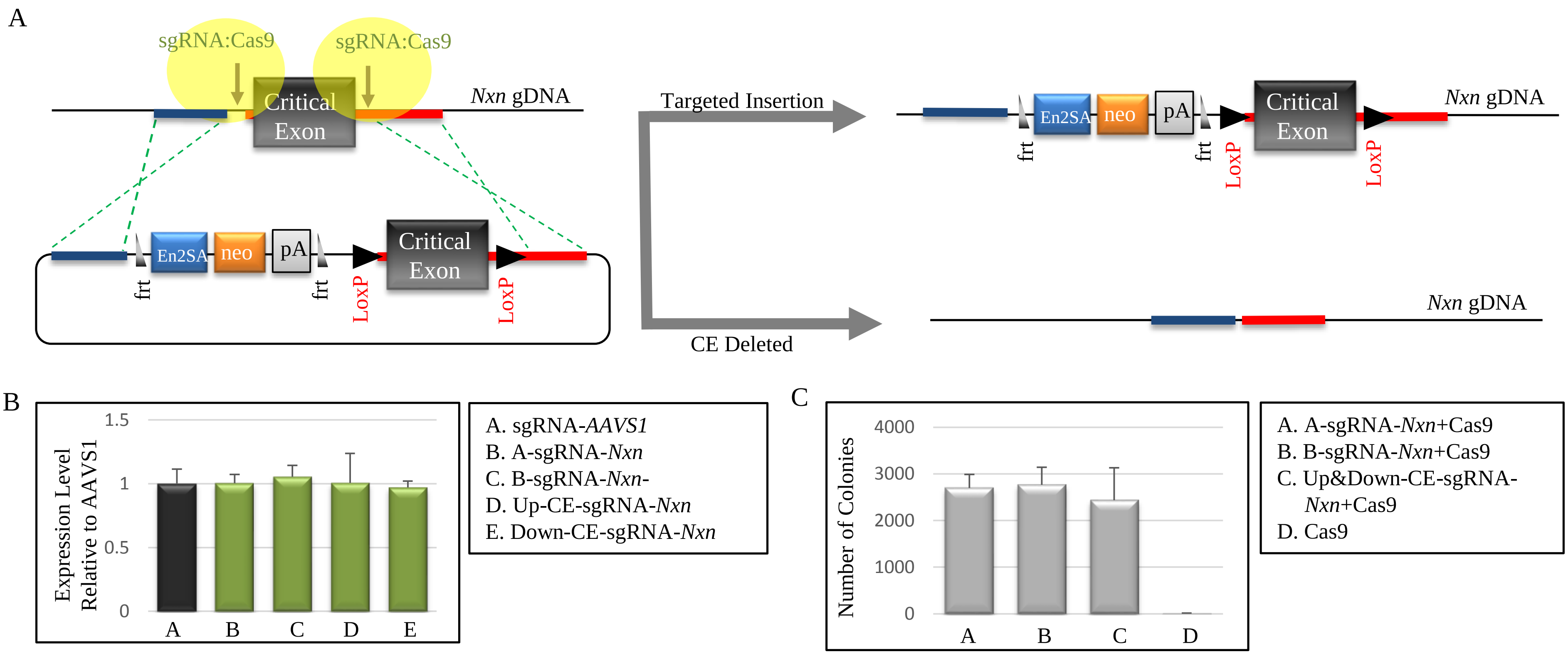

Figure 6. Strategy for CRISPR/Cas9 Mediated Multi-Vector Targeting via HDR and NHEJ Induced Gene Knockout.
(A) Schematic diagram illustrating strategy for vector targeting and deleting critical exons with NHEJ. sgRNAs (downward arrows) were designed such that the sgRNA:Cas9 complexes (yellow circle) induce dsDNA cleavage flanking the critical exon. The sgRNA complementary sequences were replaced with loxP sequences in the targeting vector. This approach may lead to two potential outcomes for which either the construct is properly targeted or the critical exon is deleted due to a repair mechanism initiated by NHEJ.
(B) Bar graph showing SSA assay of Upstream(Up)-CE-sgRNA-*Nxn* and Downstream(Down)-CE-sgRNA-*Nxn* relative to sgRNA-*AAVS1*, A-sgRNA-*Nxn*, and B-sgRNA-*Nxn*.
(C) Bar graph showing the number of G418-resistant colonies containing plasmids expressing Up- and Downstream-CE-*Nxn*-sgRNAs and Cas9 along with pKOMP-*Nxn*-900. Results are relative to transfections done using pKOMP-*Nxn*-900 with plasmids expressing Cas9 alone as a negative control, A-sgRNA-*Nxn*, or B-sgRNA-*Nxn*.

Supplemental Figure 7

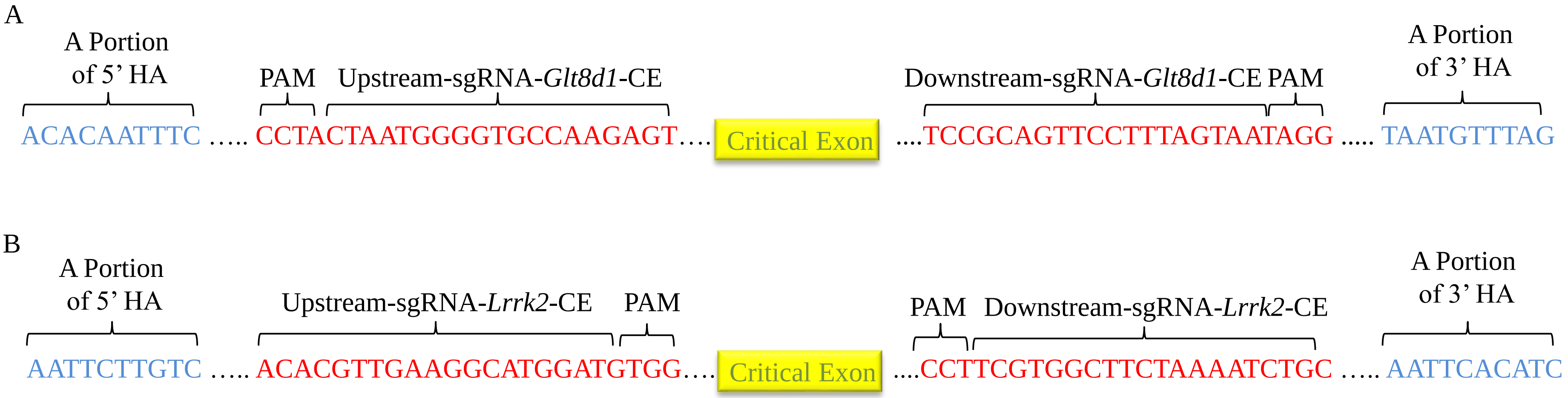

Supplemental Figure 7. sgRNA Sequences. (A) and (B) show sgRNA target sequences (red), flanking the critical exon region (yellow). Blue sequences indicate a small portion of the homology arm sequences.

Supplemental Figure 8

Genotyping Primer ID	Sequences (5 --> 3)	F/R
5' Junction for pKOMP-Nxn	TCGGCCATCATTGGAAGAGAG	F
	GGTGGTGTGGGAAAGGGTTCGAAG	R
3' Junction for pKOMP-Nxn	CTCTTCATGGTAATCAGTATGCACAGC	F
	GGGATCTCATGCTGGAGTTCTTCG	R
5' Junction for pKOMP-Nxn-900	TCTAGGCCAGTCTGGTCTACAGAGC	F
	GGTGGTGTGGGAAAGGGTTCGAAG	R
3' Junction for pKOMP-Nxn-900	CACATGATGAGATTAAAGGCATGAGC	F
	GGGATCTCATGCTGGAGTTCTTCG	R
5' Junction for pKOMP-Dbn1-900	caaggttctgagcccttctg	F
	GGTGGTGTGGGAAAGGGTTCGAAG	R
3' Junction for pKOMP-Dbn1-900	CTCTTCATGGTAATCAGTATGCACAGC	F
	cagagggagacaccagaagc	R
5' Junction for pKOMP-Asic4-900	tcaactcaagcatgcactcc	F
	GGTGGTGTGGGAAAGGGTTCGAAG	R
3' Junction for pKOMP-Asic4-900	CTCTTCATGGTAATCAGTATGCACAGC	F
	cctcaatccagcctcttcag	R
5' Junction for pKOMP-Glt8d1-900	CTT CAA CAG AGG GCC AGGAG	F
	ccg cct act gcg act ata ga	R
3' Junction for pKOMP-Glt8d1-900	gag atg gcg caa cgc aat taa tg	F
	TGG CTT TCT TTG CAC TGGGA	R
5' Junction for pKOMP-Lrrk2-900	TGT GAC GAT GGG AGA AAGCC	F
	ccg cct act gcg act ata ga	R
3' Junction for pKOMP-Lrrk2-900	gag atg gcg caa cgc aat taa tg	F
	aag cta agc agG TCa gcc ag	R
Deletion for pKOMP-Glt8d1-900	CTG CAC CCA GGG CCT TATAC	F
	GAG CCA TCA TGC CCA GTTCT	R
Deletion for pKOMP-Lrrk2-900	GTC TAC GGT TAT GGG ACTCCC	F
	TGC CTA TGT GGG GTG AAAGG	R
